# Visual encounters on line transect surveys under-detect carnivore species: Implications for assessing distribution and conservation status

**DOI:** 10.1371/journal.pone.0223922

**Published:** 2019-10-30

**Authors:** Jose M. V. Fragoso, Fernando Gonçalves, Luiz F. B. Oliveira, Han Overman, Taal Levi, Kirsten M. Silvius

**Affiliations:** 1 Stanford University, Stanford, CA, United States of America; 2 Departamento de Vertebrados, Museu Nacional, UFRJ, RJ, Brazil; 3 Environmental and Forest Biology, State University of New York-College of Environmental Science and Forestry, Syracuse, NY, United States of America; 4 Department of Fisheries and Wildlife, Oregon State University, Corvallis, OR, United States of America; 5 Department of Forest Resources and Environmental Conservation, Virginia Tech, Blacksburg, VA, United States of America; Bowling Green State University, UNITED STATES

## Abstract

We compared the distribution and occurrence of 15 carnivore species with data collected monthly over three years by trained native trackers using both sign surveys and an encounter-based, visual-distance method in a well-preserved region of southern Guyana (Amazon / Guiana Shield). We found that a rigorously applied sign-based method was sufficient to describe the status of most carnivore species populations, including rare species such as jaguar and bush dog. We also found that even when accumulation curves for direct visual encounter data reached an asymptote, customarily an indication that sufficient sampling has occurred to describe populations, animal occurrence and distribution were grossly underestimated relative to the results of sign data. While other researchers have also found that sign are better than encounters or camera traps for large felids, our results are important in documenting the failure of even intensive levels of effort to raise encounter rates sufficiently to enable statistical analysis, and in describing the relationship between encounter and sign data for an entire community of carnivores including felids, canids, procyonids, and mustelids.

## Introduction

Carnivores have a special place in the human imagination [1] and are of great ecological interest due to their functional role as drivers of top-down trophic cascades and regulators of ecosystem processes [2,3,4]. For these reasons, global conservation strategies have often focused on carnivores [5,6], defining them as flagship, keystone, or umbrella species [7]. Successful conservation and management of these predators (as well as other vertebrates) requires accurate knowledge of range area and occurrence patterns and practical methods of data analysis. Multiple methods have been developed to analyze and model field-based data and establish the conservation status of the species, including occupancy and detectability methods [8,9] and species distribution models [10,11,12]). The results of these analyses, however, depend strongly on the original quality of the field data. Ignoring imperfect detection of animals in the field can bias estimates of occupancy and related parameters, which results in misleading inferences about the system [13,14,15,16] and inappropriate management decisions.

Line transect surveys of direct visual encounters (henceforth “encounters”) have been the preferred method for collecting data on the distribution and abundance of large vertebrates for decades [17]. The approach continues to be widely employed in tropical countries (e.g., [18,19,20,21,22]) and is especially useful in participatory and citizen science programs, where local non-scientists monitor wildlife populations [23,24,25]. In this context the method is favored because it has few equipment and technology needs, is easy to learn and low in cost [26,17].

Direct encounter surveys can however be rendered impractical by low population densities, wariness of animals, nocturnality, dense vegetation or difficult terrain [27,28,19]. The method under-detects or fails to detect cryptic, shy and nocturnal species, and species that have changed their behavior due to persistent hunting or other human disturbance, [29,30,31,17], a behavioral response common to most carnivores. Failure to detect animals can lead to underestimates of animal occurrence, abundance, density and range [32,33,34]. This failure of the encounter method has been a concern when studying carnivores in Australia [35], North America [32], Europe [36], Africa [37], Asia [38] and South America [39,17].

Camera trapping and track or sign-based surveys are alternative methods for quantifying the abundance and distribution of rare or cryptic species [39,40]. Field-deployed, self-triggering cameras require no special wildlife detection skills and are constantly ready; when properly set up they should therefore detect the targeted species [41]. Cameras do in fact improve the quality of presence and abundance data for targeted species [40,42,8]. However, their expense; limited ability for deployment over landscape scales; susceptibility to theft or destruction in areas with hunters and other humans; unwieldiness to deployment in large numbers due to weight; inability to detect arboreal species without special placement; requirement for specialized technical skills for camera maintenance; and, more recently, a need for computer processing of photos [41], all limit their usefulness for biodiversity or multi-species surveys in remote regions, over large areas and by people lacking technical skills [43,44,45,46,47,31].

Indirect population assessment techniques using tracks, feces, burrows and other sign of animals have a long history of use for assessing and monitoring vertebrate populations [48], including carnivores [1]. However, the reliability of this approach has often been questioned on the basis of the similarity of appearance of tracks of different species, variance in the probability of detecting tracks on different substrates in different seasons and variance in the identification abilities of human trackers [49,50,51,52]. In response to this criticism and a need to improve the reliability, accuracy and simplicity of field wildlife monitoring methods, Fragoso and colleagues [17] developed a rigorous sampling protocol for animal sign and deployed it in a region of tropical forests, savannas, wetlands and woodlands in a Guiana Shield—Amazonian region of Guyana, covering an area about the size of Costa Rica. They successfully measured the presence, distribution and relative abundance of 32 large vertebrate species, including the 16 most heavily hunted (non-carnivore) species in their study area. They found their sign method to be superior to the encounter method for detecting and estimating occurrence and distribution of cryptic, shy, nocturnal and hunted species.

Here we use the same data set referenced in the Fragoso et al. [17] study but focus on the carnivore species in the study region, a group not previously analyzed. We used the encounter and indirect sign-based Fragoso transect protocol to measure the occurrence and distribution of 15 mostly rare or rarely seen carnivore species. We hypothesized that 1) carnivores would be better detected using the sign-protocol, 2) sign and encounter data would be correlated and 3) all species would occur and be widely distributed in this relatively undisturbed region. We also provide estimates of the distances that must be surveyed to obtain reliable estimates of carnivore occurrence for all species, with a more detailed examination for the jaguar (all scientific names in Table 1).

**Table 1:** Number of transects (out of a possible total of 216) on which carnivore species were observed by sign or encountered visually and correlations between number of carnivore visual and sign encounters *on transects where both occur*, after 12–38 resampling events (8 transects each around 23 indigenous communities and 4 uninhabited sites). Data collected from April 2007 to June 2010. r = Pearson correlation coefficient.

Common	Scientific	Relative Detection	Transects with	Transects with	Failure to	Pr, r value	Pr, P value
Name	Name	Sign vs. Visual Encounter	Encounters	Sign	Encounter		
Felidae							
Cougar	Puma concolor	Sign	10	90	80 (88.8%)	0.429	0.0014
Jaguar	Panthera onca	Sign	18	105	87 (82.8%)	0.553	0.0007
Jaguarundi	Herpailurus yagouaroundi	Sign	4	8	4 (50%)	0.514	0.0006
Ocelot	Leopardus pardalis	Sign	12	55	43 (78.1%)	0.356	0.0019
Oncilla	Leopardus tigrinus	Sign	8	20	12 (60%)	0.592	0.0006
Margay cat	Leopardus wiedii	Sign	5	40	35 (87.5%)	0.537	0.0008
Mustelidae							
Giant otter	Pteronura brasiliensis	Sign	5	12	7 (58.3%)	0.331	0.0021
Neotropical otter	Lontra longicaudis	Sign	8	12	4 (33.3%)	0.608	0.0107
Tayra	Eira barbara	Visual Encounter	64	28	36 (56%)*	0.198	0.0686
Grison	Galictis vittata	Sign	5	18	13 (72.2%)	0.548	0.0008
Procyonidae							
Kinkajou	Potos flavus	Equally	5	5	0	0.236	0.0170
Coati	Nasua nasua	Equally	57	57	0	0.460	0.0402
Crab-eating raccoon	Procyon cancrivorus	Sign	5	90	85 (94.4%)	0.640	0.0081
Canidae							
Bush dog	Speothos venaticus	Sign	10	20	10 (50%)	0.576	0.0006
Crab-eating fox	Cerdocyon thous	Sign	55	110	55 (50%)	0.474	0.0026

## Materials and methods

### Study area

**Fig 1:**
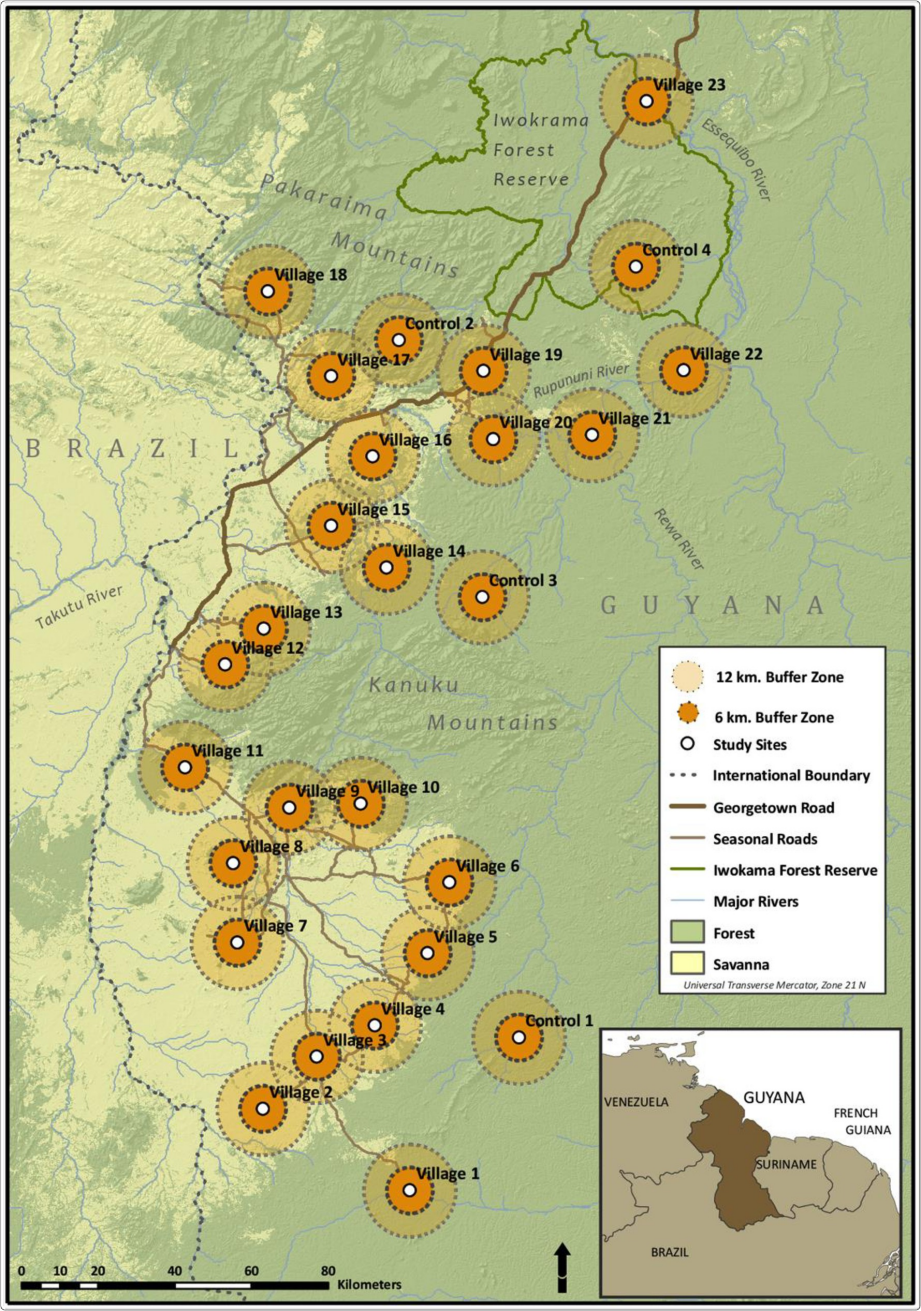
Study area in southwest Guyana study region showing village sites and non-village (marked as Control in figure) sites and the 0–12 km distance zones for transect placement (map adapted from [17]).

The study took place in Region 9 of Guyana (2.5167 Latitude; -59.2499 Longitude), commonly known as the Rupununi (Fig 1). The study region encompasses approximately 48,000 km^2^ and includes continuous old growth lowland and mountain forest, woodlands (similar to the Brazilian *cerrado*), upland and flooded savannas, forest islands, and gallery forest [53]. Mountains covered by dry and wet forests border the region to the north and south. Elevation ranges from 1,100 m above sea level in the Pakaraima and Kanuku mountains to 30 m in lowland savanna and swamp areas (Fig 1). Rainfall occurs predominantly during one rainy season from May to September [54]. The study area included most of the territories of 20,000 Makushi and Wapishana people [24]; village size in the area ranged from 122 to 1192 inhabitants [17]. The predominant livelihood in all villages is subsistence hunting, fishing and farming. No large-scale habitat degradation exists in the study area; a few villages practice small-scale selective timber extraction for local use. Detailed vegetation classification along transects is described elsewhere [53]. The project was permitted by the Guyana Environmental Protection Agency (EPA Permit: 171106 BR 064).

### Encounter and sign sampling design

To capture a full range of sign and encounter data, we use the encounter and indirect sign-based Fragoso transect protocol [17]. Eight straight-line, 4 km-long transects were established around 23 indigenous communities and 4 uninhabited sites (henceforth village and non-village sites), for a total of 216 transects [17]. Transect placement was randomized within distance bands of 0–6 km and 6–12 km from the village center (or site center in the case of non-village sites); a 3 km minimum distance was maintained between transects to maximize the independence of individuals detected. In areas where different villages’ 12 km concentric areas overlapped, transect placement was stratified to avoid placement in the overlap zone. In rare situations when impassable barriers were encountered (e.g., a cliff) that could not safely be traversed, the transect extended in the opposite direction to its 4 km end point and if this was not possible, it turned instead to the right at a 90° angle and continued until the 4 km end point. Transect length was selected to increase the probability that transects would traverse the home range of the farthest ranging species in the system, including cougar and jaguar.

Encounter and sign data require different sampling techniques and were therefore collected at different times on the same transects. Animal sign (predominantly tracks, but also feces, hair, carcasses, body parts, digging, burrows, markings, and partly eaten fruits or seeds) and direct encounter data were collected monthly (with two weeks separation between methods) by indigenous trackers trained in distance sampling methodology [24] from May 2007 to June 2010. Due to the large sample size and timing of transect implementation, the 27 study sites were incorporated to the study in a time-staggered fashion, such that by the end of the study individual sites had a minimum of 12 and a maximum of 38 months of data collection (median = 27 months). Cumulative distance walked on the 216 transects by the end of the study was greater than 50,000 km. For encounter data, we used standard distance sampling methods [55]. To avoid recounting the same sign during subsequent monthly sampling periods, only sign deemed by indigenous trackers to have been left within the three days preceding sampling were recorded. Sign data were collected only within a one-meter center width of the entire transect. Collection of sign and encounter data on the same transects within two weeks of each other ensured that identical vegetation and substrates were sampled by both techniques, eliminating this as a possible confounding variable. To check for commonly made recording errors, we used standardized check sheets during each monthly data collection session. For each parabiologist we also reviewed data collection reports validated by the community leader in each village each month (see Luzar et al. [24] for a deeper discussion of data quality control). We followed Wilson and Reeder [56] for the taxonomic classification and behavior description of the carnivore species surveyed.

We calculated the *effectiveness* of each method in detecting a species on transects where it was known to be present as the percentage of those transects where it was detected by the best method where it was also detected by the worst method. Although all sign and encounters of species were recorded on each transect during each sampling event, for this analysis a single detection event for sign or encounter determined species occurrence on a transect.

To compare the *efficiency* (level of effort required) of sign and encounters in detecting species occurrence, we constructed data accumulation curves for each method for all species. Accumulation curves represent the number of transects on which a species is detected relative to the number of times that the study area (all transects) is re-sampled. The number of walks (re-sampling events) required before the curve reaches an asymptote (customarily considered a sign that sufficient sampling has occurred to describe populations) indicates the level of effort necessary to maximize detections with each method. To calculate the effort (walked distance) needed to attain an asymptote in each method, we multiplied 216 transects (total) by length (4 km-long) by effort (number of walks) needed to asymptote. This estimate of walk effort is conservatively biased low because some fraction of transects where resurveying was halted prematurely may have eventually detected a focal species, which could allow the accumulation curve to asymptote at a higher number of resampling events. However, this effect is expected to be small because 79% of transects were surveyed at least 20 times and 85% at least 15 times.

To assess the potential for sign data to replace encounter data as a quantitative measure of abundance, we calculated Pearson’s correlation coefficients between the total number of visual encounters and total number of signs detected over the course of the study on each of the transects where carnivore species were detected by both methods. We used a paired (sign versus visual encounter) comparison on the same transect where both occur.

In all analyses, transects from village and non-village sites were combined.

## Results

### Occurrence and distribution

Sign data performed better than encounter data in detecting 12 carnivore species, equally well for 1 species, and less well for 2 species (Table 1). The encounter method failed to detect 13 of 15 species at 34–48% of sites (village and non-village sites combined) where they were confirmed to occur on the basis of sign data. At the transect level, the encounter method failed to detect these carnivores on 33–89% of transects where they were detected by sign (Table 1).

#### Felidae

All felids had higher detection rates with sign than with encounters (Table 1). Failure to detect by encounter during diurnal transects was, as expected, high (50 to 88.8%) for cougar, jaguar, ocelot, oncilla, jaguarundi and margay cat. The cougar had the highest failure-to-encounter rate (88.8%; 80 of 90 transects), followed by the margay cat (87.5%; 35 of 40 transects) (Table 1). Nevertheless, on transects where a felid species was detected by both methods, the frequencies of encounter derived from each method were positively correlated for all species (Pearson’ s correlation coefficient, r = 0.356 to 0.592, p = 0.0006 to 0.0019; Table 2).

**Table 2:** Effort needed for accumulation curve of best detection method (Sign, Visual Encounter) to reach asymptote for 15 carnivore species. (T = terrestrial, S-A = semi-aquatic, A = arboreal, N = nocturnal, D = diurnal, C = crepuscular).

Taxa	Relative Detection	Number of transects	Effort needed	Walk-distance needed	Habit
	Sign vs. Visual Encounter	needed to Asymptote	to Asymptote (N walks)	to Asymptote (Km)	
Felidae					
Cougar	Sign	78	28	24,192	T, N & D
Jaguar	Sign	105	26	22,464	T, N & D
Jaguarundi	Sign	8	25	21,600	T & D
Ocelot	Sign	53	33	28,512	T & N
Oncila	Sign	20	29	25,056	T & N
Margay cat	Sign	40	28	24,192	T & A, C
Mustelidae					
Giant otter	Sign	12	26	22,464	W
Neotropical otter	Sign	12	26	22,464	W
Tayra	Visual Encounter	28	25	21,600	T, A & D
Grison	Sign	15	20	17,280	T & A
Procyonidae					
Kinkajou	Visual Encounter *	5	12	10,368	A
Coati	Equally	65	30	25,920	T, A & D
Crab-eating raccoon	Sign	90	25	21,600	T
Canidae					
Bush dog	Sign	20	22	19,008	T
Crab-eating fox	Sign	100	10	8,640	T

*both the encounter and sign accumulation curve reached an asymptote at the same number of transects, but encounters attained this with fewer walks

#### Mustelidae

Two semi-aquatic and one terrestrial mustelid (giant otter, Neotropical otter and grison) had higher detection rates with sign than encounters (Table 1), with the grison exhibiting the highest failure-to-encounter rates 72.2% (13 of 18 transects) followed by giant otter 58.3% (7 of 12 transects) (Table 1). In contrast, the scansorial tayra was more frequently detected by encounters 56% (64 transects) than sign (28 transects) (Table 1). On transects where a mustelid species was detected by both methods, the frequencies of detection derived from each method were positively correlated for giant otter, Neotropical otter and grison (r = 0.331 to 0.608, p = 0.0008 to 0.0107) but not for tayra (r = 0.198 p = 0.0686; Table 2).

#### Procyonidae

Sign data performed better or equally as well as encounter data for procyonids. The two terrestrial and one arboreal procyonid—crab-eating raccoon, coati, and kinkajou—had higher or equal detection rates with sign (Table 1); the crab-eating raccoon had the highest failure-to-encounter rate (94.4%; 85 of 90 transects) while the coati and kinkajou had zero failure-to-encounter rates (0 out of 57 and 5 transects respectively) (Table 1). On transects where a procyonid species was detected by both methods, the encounter frequencies derived from each method were positively correlated for all species (r = 0.236 to 0.640; p = 0.0081 to 0.0402; Table 2).

#### Canidae

Sign data performed better than encounter data for detecting the two canid species found in the area. The crab-eating fox (55 of 110 transects) had equal failure-to-encounter rates (50%) as the bush dog (10 of 20 transects) (Table 1). On transects where a canid species was detected by both methods, the frequencies of encounter derived from each method were positively correlated (r = 0.474 and 0.576; p = 0.0026 and 0.0006 for crab-eating fox and bush dog respectively; Table 2).

### Accumulation curves

**Fig 2:**
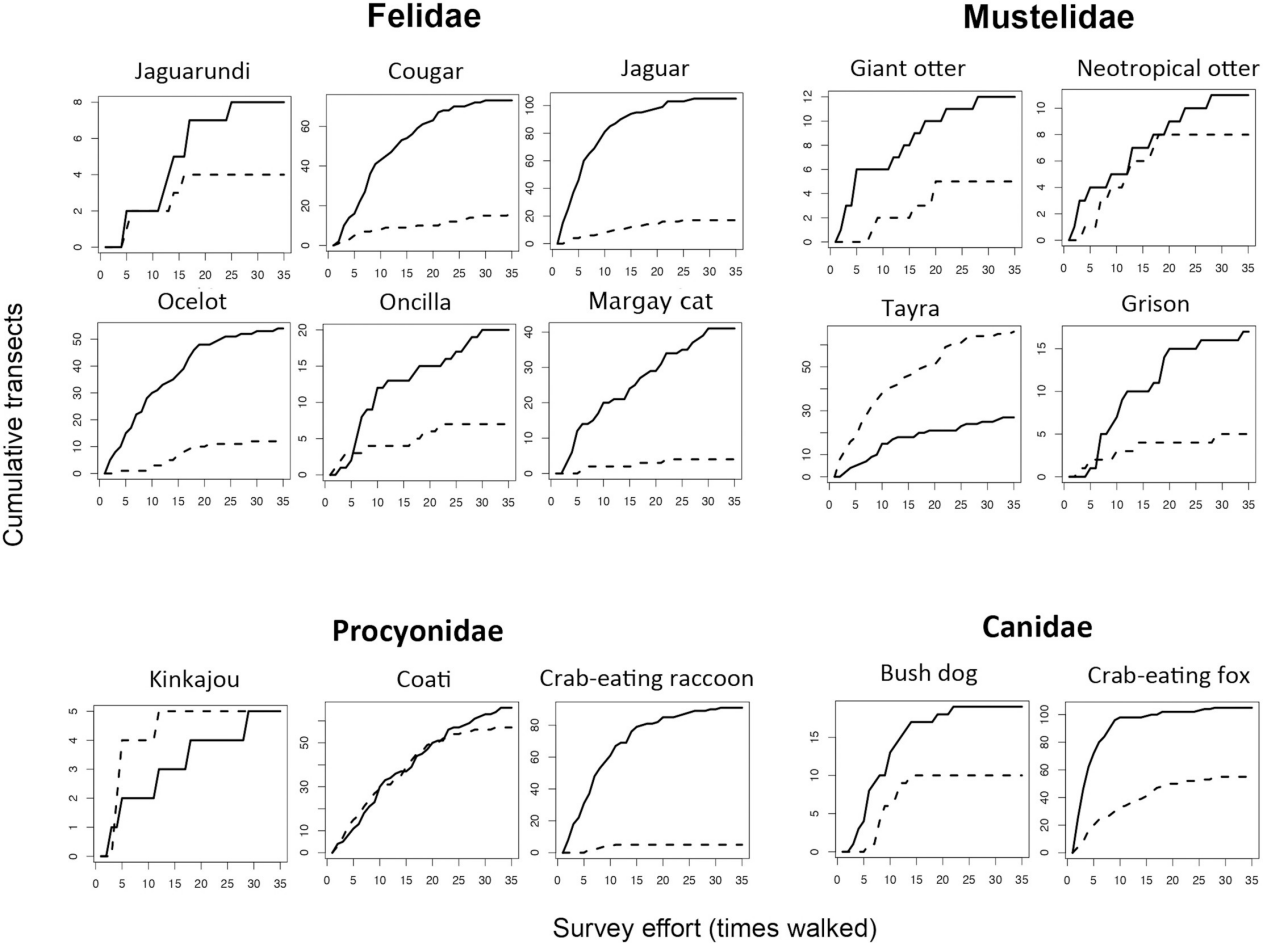
Accumulation curves for sign vs. encounters (number of transects on which species was detected at least once as the study progresses) for 15 carnivore species. Effort is reported in terms of survey months and equivalent kilometers walked. The Y-axis represents the number of transects with presence recorded. The X-axis represents the number of times the same transects were walked (i.e., number of times you have to resample the study area). Solid lines represent sign and the hatched lines encounters.

For all 15 carnivore species, the number of transects walked during the study was sufficient for the accumulation curve to reach an asymptote with at least one of the methods (Fig 2 and Table 2), but the number of transects on which a species had been detected when the curve attained an asymptote differed between the two methods. For 12 of 15 species, sign accumulation curves reached an asymptote with more transects and fewer or similar number of walks than the encounter accumulation curves, supporting the hypothesis that sign is the more efficient and effective method for describing carnivore species occurrence and distribution (Fig 2 and Table 2). For the coati, encounters and sign performed similarly well at capturing the species’ distribution (Fig 2 and Table 2). For the arboreal kinkajou, both the encounter and sign accumulation curve reached an asymptote; encounters attained this with fewer walks at the same number of transects (Fig 2). In contrast, for the tayra encounters achieved an asymptote with more transects than the sign accumulation curve.

### Walking distance required for sufficient detection

Cougar and jaguar required 24,192 (216 transects x 4 km x 28 walks) and 22,464 (216 transects x 4 km x 26 walks) km respectively of distance walked for the sign accumulation curves to reach their asymptotes (Table 2). For these species, encounter accumulation curves reached asymptotes after slightly less sampling effort, but the animals were detected on fewer transects at asymptote than with sign: 10 (vs 78) and 18 (vs 105) transects with 26 (vs 28) and 21 (vs. 26) walks for cougar and jaguar, respectively (Fig 2).

Sign accumulation curves for smaller felids reached asymptotes at 8 (jaguarundi), 53 (ocelot), 20 (oncilla) and 40 (margay cat) transects after 25, 33, 29 and 28 walks, respectively, equivalent to 21,600 (jaguarundi), 28,512 (ocelot), 25,056 (oncilla) and 24,192 km (margay cat) (Fig 2 and Table 2). For all these species, encounter accumulation curves required fewer or similar walks (equaling shorter or same distances) to reach asymptote, but animals had been detected on fewer transects at that point.

For the giant and Neotropical otters, sign accumulation curves attained an asymptote on the same number of transects (12) with the same number of walks (26), equaling a total distance of 22,464 km. The grison curve reached an asymptote on 15 transects at 20 walks with sign data, an equivalent of 17,280 km of walked-distance. For otters and grison, sign data outperformed encounter data. For tayra, in contrast, encounter curves reached asymptotes at more transects and with fewer walks than sign curves, requiring 21,600 km of distance walked versus 30,415 km for sign (Fig 2 and Table 2).

Among the procyonids, the sign accumulation curves for the crab-eating raccoon achieved an asymptote at 90 transects after 25 walks (21,600 km of walked-distance). For kinkajous, the encounters accumulation curve reached an asymptote at 5 transects walked 12 times (10,368 km walked), while sign attained an asymptote on 5 transects only after 30 walks (26,920 km walked). Encounters and sign perform similarly well for coati, requiring 25,920 km of walked-distance for the accumulation curves to reach asymptotes (Fig 2 and Table 2).

Sign data performed better than encounter data for the two canids with curves reaching asymptotes at 100 transects for the crab-eating fox and 20 for the bush dog, after 10 to 22 walks and 8,640 and 19,008 km of walked-distance, respectively (Fig 2 and Table 2).

### Jaguar and lack of detection

**Fig 3:**
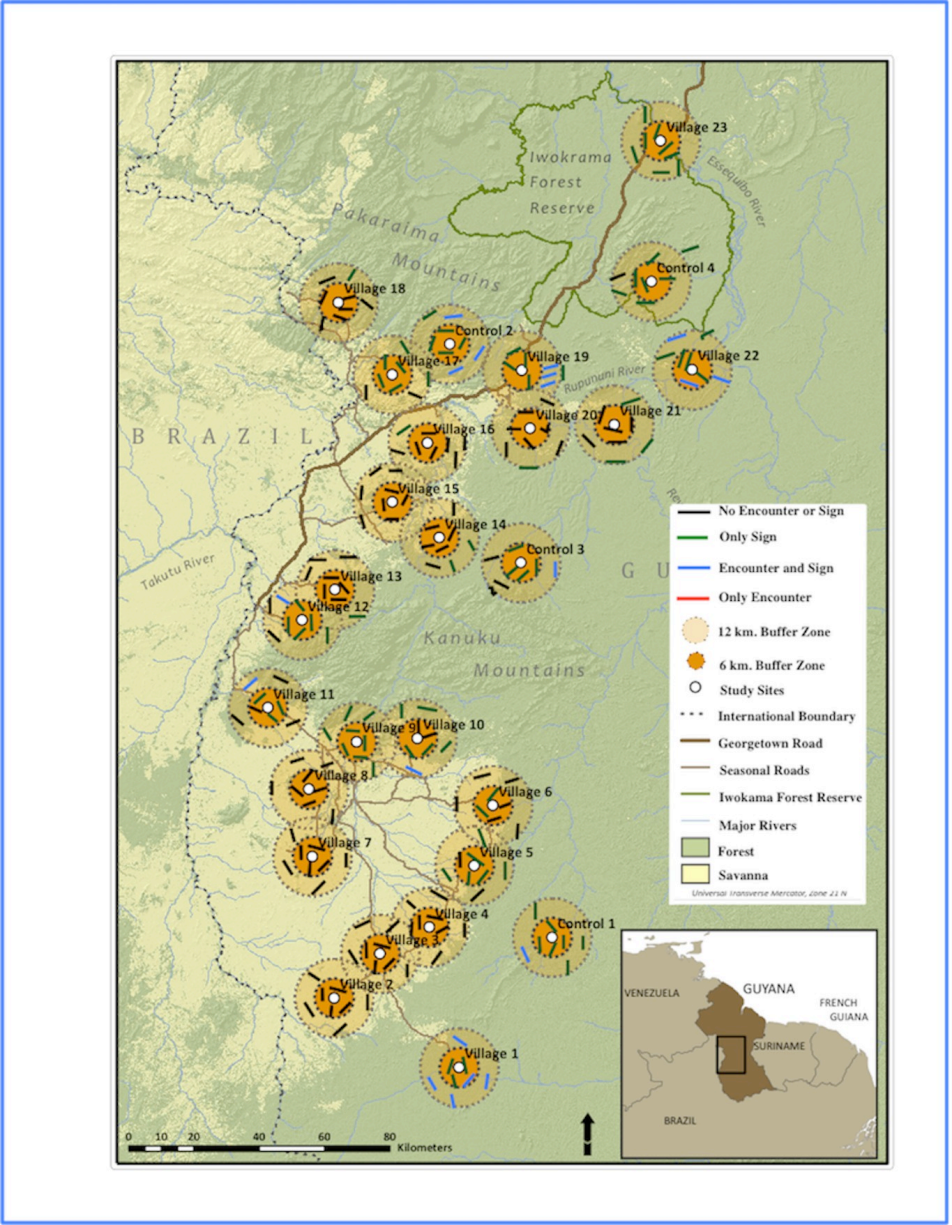
Jaguar occurrence on transects around northern indigenous village and un inhabited sites (marked as Control in figure) study sites in Region 9, Guyana (map adapted from [17]).

The jaguar was the most hunted carnivore in the area, with 22 individuals killed across 23 communities (with a total human population 9352) over 3.5 years [17]. The species was detected by sign around 17 of 23 villages and at all four sites unoccupied by humans but was visually encountered at only 6 of these villages and 3 of the sites unoccupied by humans (Fig 3). It was detected by sign on 105 transects, but never directly encountered on 87 of those same transects (Fig 3).

## Discussion

The availability of reliable, accurate and cost-effective methods for monitoring vertebrate populations remains a critical need in wildlife biology. In our comparison of encounter and sign-based detection methods for carnivores, we found that the encounter-based method often failed to detect animals at sites where the sign data confirmed them to be present. Sign surveys may be the most viable method for large-scale, management-oriented studies in remote tropical areas where funding is limited, particularly those focused on community-based wildlife management [24,26]. Sign-based methods can identify areas intensively used by species of interest, which should be prioritized for protection and management [57,58]. Similar findings have been made at other tropical areas (e.g., [59,39,26]). Our study is unique in validating this finding with three years of effort, which allowed us to produce robust accumulation curves (Fig 2), across the entire community of carnivores at a landscape scale.

### Efficiency and effectiveness of sign vs. encounter-based transects

We hypothesized that carnivores would be better detected and counted using the sign-based protocol developed by Fragoso and colleagues [17] than by direct encounters. This was true for 12 of the 15 species of carnivores in our study area. For the diurnal—scansorial coati and the nocturnal—arboreal kinkajou both methods performed equally well, while for the diurnal—scansorial tayra direct encounters were more effective. Fragoso et al. [17] obtained similar results for non-carnivores, showing that 14 heavily hunted terrestrial species were best detected by sign, while 8 lightly hunted or unhunted species of arboreal primate and 2 species of hunted large birds were better detected by direct encounters.

The similarity in encounter and sign data results for coati may be due the lack of hiding behavior in this species [60], similar to the finding for agoutis in the same study area [17]. For terrestrial crepuscular or nocturnal carnivore species, on the other hand, the sign method performed better for detection than the encounter methods. Jaguars were occasionally killed as pests in the study area, and hiding behavior added to nocturnal habits may further reduce the effectiveness of the encounter method [61]. Others have found that in areas where they are not hunted, jaguars are most often detected during daylight hours [61].

Achieving an asymptote in the data is considered a reliable approach for determining that sufficient sampling has occurred to provide a realistic representation of species occurrence and/or abundance [62,63]. Surprisingly, we found that with the encounter method, accumulation curves reached asymptotes at fewer transects and after fewer walks than sign accumulation curves. For example, the jaguar sign accumulation curve reached an asymptote only after the species had been detected on 105 transects, which required 216 transects to be walked 26 times. In contrast, encounter data accumulation curves reached asymptote after the species had been detected on just 18 transects after walking 216 transects 21 times. In this case reaching an asymptote at lower effort is not an indicator of efficiency or accuracy of the method, as the encounter method grossly underestimated the occurrence and distribution of jaguars, which were in fact present on an additional 87 transects. If accumulations curves reach asymptote before jaguars are detected at all sites where they are present, then the number of transects and times they were walked is insufficient to calculate distribution and occurrence. This was true for 12 of the species we sampled, indicating that for these animals’ density, distribution and possibly occupancy estimates based on encounters will produce gross underestimates of actual occurrences.

The above result challenges the assumption that an asymptote in data indicates that an adequate amount of sampling has occurred to represent the status of a population. As with our study, efforts with carnivores in North American [32], Europe [36], Africa [37] and Asia [38] have described a similar under-detection of carnivores using standard sampling methods. These authors also recommended using a mix of approaches if sampling a variety of species, and no visually based methods when sampling species that are impacted by human activities.

Obtaining a statistically reasonable number of observations with either sign or encounter data to calculate occurrences for rarely encountered animals required extensive sampling effort. For jaguars, we walked 22,464 km for sign and 18,144 km for encounters before data reached an asymptote. Both these distances are far greater than those presented in most studies that estimated jaguar occurrence in a similar ecosystem. For example, Hill et al. [64] walked 1,426 km, Munari et al. [39] walked 343.8 km, and Carrilo [65] walked 123 km.

All carnivores in our area were unhunted or lightly hunted and only as pests (exception coati occasionally killed as food). In areas where hunting is more intensive, additional sampling effort many be required before curves reach an asymptote, due to hiding behavior by hunted animals. Shifts in behavior in response to anthropogenic impact are extensively documented for many carnivore species (e.g., [66, 67,68,10]). Factors other than hunting and human disturbances that affect encounter rates include abundance levels, naturally cryptic behavior, and observer-environment interaction. An important consideration in method selection is also the efficiency (more rapid data accumulation) of sign sampling over encounters, which would enable occurrence estimates for species that occur at low densities (e.g., bush dog) or exhibit cryptic behavior (e.g., cougar and jaguar). In our study, the sign method was most efficient for the greatest number of carnivore species.

Some general concerns with use of sign for examining vertebrate species and populations are: 1) field researchers with little or no tracking expertise can miss seeing sign (the use of expert trackers and hunters [24,26]) and searching only a 1 m wide band along the transect can reduce this problem); (2) variance can occur between trackers in ability to identify sign (training trackers as a group should align sign-species identification, along with use of a sign guide [24]), (3) false positives or false negatives can occur (the use of experts can reduce the importance of this problem but it is unlikely to be completely eliminated [24,26]); (4) availability of appropriate soil substrates for tracking (this issue can be reduced by simultaneously recording other animal sign such as dung [21] as well as tracks), and (5) variance in performance due to weariness over long tracking periods.

Despite the above concerns with using sign to asses animal populations, we found statistically significant positive correlations between sign and encounter data for 14 of 15 carnivore species, demonstrating the robust nature of Fragoso and colleagues’ [17] sign sampling protocol. Fragoso and colleagues also reported significant positive correlations between sign and encounters for the 10 most heavily hunted large vertebrates in their study. Such correlations indicate that with more effort sign could be translated into animal abundance and density, including for areas where they are never sighted, as occurs with dung counts for many species [21]. In the Kalahari region of Botswana use of body-mass day-range scaling rules allowed the conversion of track counts to densities for several difficult to observe vertebrate species [69]. A comprehensive evaluation of the relationship between track counts and animal density derived from simulations of virtual and empirical animal track and density data, verified that track number is directly determined by the density and daily movement distances of vertebrates [70]. Refining the sign approach in the Neotropics to allow calculation of animal abundance would facilitate management decisions, especially at local and regional levels. Encounter data alone should not be used because for most carnivore (and hunted) species many transects yielded no encounters but sustained individuals (as shown by sign data).

### Carnivore distribution and status

All species of mammalian carnivores previously known to occur in the region were recorded. The most widely distributed species were the large felids (jaguar and cougar), and the crab-eating raccoon and crab-eating fox. The large body size of these felids, their adaptable feeding habits and non-specific habitat requirements may explain their wide distribution over transects in a variety of vegetation types (e.g., forests, woodlands, wetlands and savanna) [71]. Their wide occurrence in our region also suggests that humans have had little impact on these species. More restricted distributions occurred for the mid-sized carnivores. This may reflect more specialized habitat requirements and/or more restricted scales of movement, preventing them from living in some habitats (e.g., margays require forests, otters require water bodies) [71]. For some terrestrial species such as the grison, bush dog and oncilla, with very restricted distributions and few detections, there is too little information from the wild to permit speculation. Kinkajous are nocturnal and arboreal and their rarity in our data set is explained by our diurnal sampling.

### Management implications

The management implications of appropriate method selection are illustrated by the case of the jaguar in this study. Mapping of the transect data for jaguars (Fig 3) illustrates the mesoscale gaps in distribution that result from encounter data and affect our understanding of population structure and hunting impacts. A conclusion of local jaguar extirpation would have been reached at 17 villages and one uninhabited site with no jaguar encounters after three years of sampling, if distribution or occurrence were determined on the basis of encounters only and sign had not been recorded. Given the location of some of these villages within or adjacent to a multiple-use protected area (e.g., Iwokrama Forest), encounter-based results pointing to the absence of jaguars could result in restrictions on sustainable resource extraction by communities and other stakeholders; for example, people could be restricted from hunting game animals for their own food in order to provide prey for jaguars. A study with camera trapping conducted in the Iwokrama Forest, which included one village and one control site from our study, validated our results for sign: it found healthy jaguar populations, with densities derived from combined camera trapping in unlogged and reduced-impact-logging locations falling within the density ranges reported in the literature for protected areas [72].

## Conclusions

Due to human activities, Neotropical carnivore species densities have declined drastically in most remaining forests outside of the Amazon region [73]. Our work indicates that direct detection methods are unreliable for occurrence and distribution estimation, at least for terrestrial or mostly terrestrial species and some scansorial and arboreal species. Logistical issues (e.g., cost, technical expertise, maintenance, lab requirements) in most tropical countries preclude the application of standard animal sampling methods, such as capture–recapture sampling, camera-traps, or-e-DNA analyses to estimate occurrence, distribution and abundance. Thus, there is a premium in developing or refining protocols that directly address or overcome the issue of imperfect detections.

We conclude that systematic, transect-based collection of sign data conducted by expert local trackers as per the Fragoso et al. method [17] is an efficient and effective method of monitoring carnivore species populations that complements or replaces encounter methods and camera traps in both well-preserved and human-impacted areas. Even with massive sampling effort, it was not possible to increase encounter-based detectability for 12 of 15 Neotropical carnivores to levels that reflect their true distribution or even occurrence; for these species, sign-based methods more reliably revealed the presence of animals and hold promise as practicable indices—or even direct measures—of abundance and density. In other cases, such as for the kinkajou, both methods similarly describe the distribution of animals on the landscape, with each method missing the species on only a few transects where it was detected by the other method (Fig 2). The decision of which method(s) to use for Neotropical carnivore surveys or monitoring of anthropogenic impacts will depend on the focal species’ habits (arboreal vs. terrestrial, diurnal vs. nocturnal, behaviorally responsive to hunting or not).

Our results and those of others (e.g., [59,39,26]) have shown that sign surveys may be the most efficient and most engaging method from a local community perspective. The resulting information can help the communities better understand and manage wildlife and livelihood dynamics in their territories. Skills gained and work history accumulated enable them to access other work opportunities in the future (e.g., with research projects, NGOs, government surveys), as well as to achieve higher decision-making status in their communities as resident experts about the community’s natural resource base [24]. The advantages of our transect-based sign survey protocol thus include accuracy of species identification, low environmental disturbance, similar efficiency in the detection of nocturnal and diurnal species (when compared with direct counts), additional possibility of studying activity patterns, ease of use by non-scientists, positive engagement with rural people and extent of area that can be simultaneously sampled.
